# Plant vernalization proteins contain unusual PHD superdomains without histone H3 binding activity

**DOI:** 10.1016/j.jbc.2022.102540

**Published:** 2022-09-27

**Authors:** Elsa Franco-Echevarría, Trevor J. Rutherford, Marc Fiedler, Caroline Dean, Mariann Bienz

**Affiliations:** 1 MRC Laboratory of Molecular Biology, Cambridge, United Kingdom; 2 John Innes Centre, Norwich Research Park, Norwich, United Kingdom

**Keywords:** PHD finger, histone H3 tail binding, Polycomb silencing, plant vernalization, OBERON family, 4HB, four-helix bundle, HA, hemagglutinin, ITC, isothermal calorimetry

## Abstract

PHD fingers are modular domains in chromatin-associated proteins that decode the methylation status of histone H3 tails. A PHD finger signature is found in plant vernalization (VEL) proteins, which function as accessory factors of the Polycomb system to control flowering in Arabidopsis through an epigenetic silencing mechanism. It has been proposed that VEL PHD fingers bind to methylated histone H3 tails to facilitate association of the Polycomb silencing machinery with target genes. Here, we use structural analysis by X-ray crystallography to show that the VEL PHD finger forms the central module of a larger compact tripartite superdomain that also contains a zinc finger and a four-helix bundle. This PHD superdomain fold is only found in one other family, the OBERON proteins, which have multiple functions in Arabidopsis meristems to control plant growth. The putative histone-binding surface of OBERON proteins exhibits the characteristic three-pronged pocket of histone-binding PHD fingers and binds to methylated histone H3 tails. However, that of VEL PHD fingers lacks this architecture and exhibits unusually high positive surface charge. This VEL PHD superdomain neither binds to unmodified nor variously modified histone H3 tails, as demonstrated by isothermal calorimetry and NMR spectroscopy. Instead, the VEL PHD superdomain interacts with negatively charged polymers. Our evidence argues for evolution of a divergent function for the PHD superdomain in vernalization that does not involve histone tail decoding.

Plant homeodomain (PHD) fingers were discovered in Arabidopsis (1) but subsequently found throughout eukaryotes, typically in nuclear proteins associated with chromatin. They are zinc-binding domains typified by a Cys4-His-Cys3 motif with characteristically spaced cysteine and histidine residues that ligate two Zn^2+^ ions in a crossbraced fashion. In addition, the PHD signature also contains a highly conserved aromatic residue upstream of the penultimate cysteine pair (2, 3). The main structural feature of PHD fingers is a three-pronged pocket that binds to histone H3 tails in a methylation-dependent manner, engaging with lysine 4 (K4) and arginine 2 (R2) through two adjacent pockets (4, 5, 6). Anchoring of this tail is achieved by a third pocket, a deep hydrophobic cavity that binds specifically to the H3 N-terminal alanine (6). Typically, K4 pockets with a preference for histone H3 trimethylated at lysine 4 (H3K4me3) exhibit an aromatic cage (7, 8), whereas K4 pockets lined with polar residues tend to prefer binding to unmodified histone H3 tails (9, 10). R2 pockets have also evolved distinct shapes to discriminate between unmodified or methylated histone H3 tails methylated at R2 (11, 12). Recognition of methylated K9 (13) or acetylated histone H3 tails (14, 15) has also been reported, but these interactions involve binding to different PHD surfaces, outside the canonical three-pronged histone H3-binding pocket. The general principle that emerged from these studies is that the variously shaped histone-binding pockets and surfaces of individual PHD fingers empower them to decode combinations of specific histone H3 tail modifications that mark actively transcribed or repressed genes (16). In addition, some PHD fingers also bind to nonhistone proteins and thus form ternary complexes that determine transcriptional outcomes (4, 6, 17).

A classical PHD signature motif is also found in plant VEL proteins (18). During vernalization, VEL proteins associate with Polycomb Repressive Complex 2 and are required for epigenetic silencing of the Arabidopsis *FLOWERING LOCUS C (FLC*) (19). Previous evidence based on pull-down assays suggested that the PHD finger of VIN3 (VIN3_PHD_) binds to histone H3 tails bearing specific methylations (20), despite the lack of a predicted aromatic cage in its putative K4 pocket. Furthermore, a mutation that restored an aromatic cage in VIN3 enhanced its binding to histone H3K4me3 and resulted in hyper-repression of *FLC* (21). Based on their results, the authors proposed that the decoding of modified histone H3 tails at *FLC* by VIN3_PHD_ was crucial for *FLC* silencing and for the vernalization response of Arabidopsis plants (21).

We therefore decided to determine the crystal structure of VIN3_PHD_ to examine the shape of its putative histone-binding pockets. Here, we show that this PHD finger is flanked by a zinc finger (ZnF) and a four-helix bundle (4HB) and thus forms an integral part of a compact tripartite superdomain. This PHD superdomain is found only in one other family of proteins, namely the OBERON (OBE) proteins that function in the shoot and root meristems to control the growth of Arabidopsis plants (22, 23, 24, 25). However, the PHD finger modules in OBE proteins exhibit a typical three-pronged pocket architecture found in histone-binding PHD fingers and we show using isothermal calorimetry (ITC) that the purified PHD superdomain of OBE1 binds to methylated histone H3 tails, as expected from its predicted pocket architecture. In contrast, the VIN3 PHD finger is markedly different from this architecture, and its putative histone-binding surface (‘front’ face) exhibits a positively charged patch that may prevent it from binding to positively charged histone tails. Indeed, using ITC and NMR spectroscopy, we were unable to detect binding of the purified VIN3 PHD superdomain nor of its minimal PHD finger to various modified and unmodified histone H3 tail peptides. This argues against a role of VEL proteins in histone H3 decoding. We provide evidence that their PHD superdomain can interact instead with negatively charged polymers such as DNA and RNA, supporting the notion that VEL PHD superdomains bind to nonhistone tail ligands bearing predominantly negative charges.

## Results

### The VIN3 PHD finger is an integral part of a tripartite superdomain

**Figure 1:**
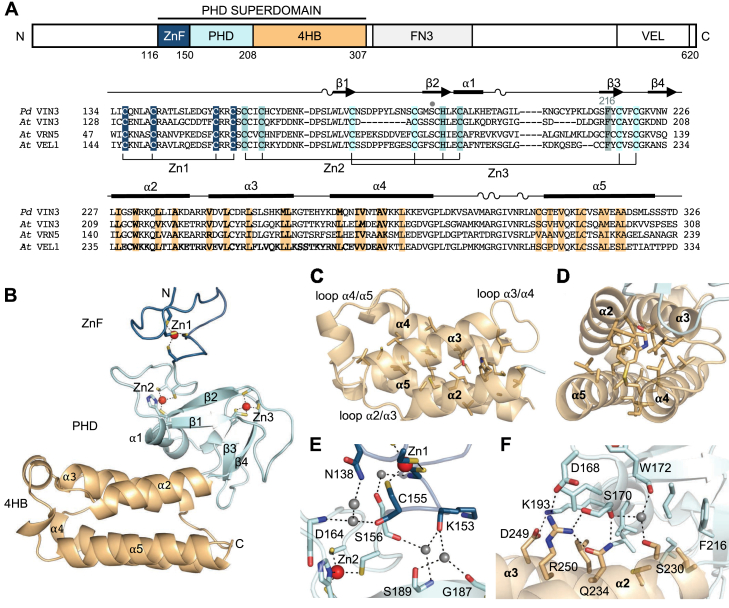
**The PHD finger of VIN3 is an integral part of a tripartite superdomain.***A*, domain architecture of *P. dactylifera* (*Pd*) and *A. thaliana* (*At*) VEL proteins (residue numbers, *At* VIN3); underneath, sequence alignment of VEL paralogs with secondary structure on *top*, as indicated; highlighted are Zn^2+^-ligating residues in ZnF (*blue*) and PHD signature residues (including Phe_216_, *cyan*) and conserved hydrophobic residues of 4HB (*light orange*); *dot*, surface-exposed serine equivalent to the fourth aromatic cage residue in K4me3-binding pockets (see also Fig. S2). *B*, overall structure of VIN3_123-326_, with N and C termini indicated; *blue*, ZnF; *cyan*, PHD; *light orange*, 4HB; *red balls*, zinc ions. *C* and *D*, hydrophobic core of 4HB, with four α-helices labeled. *E*, interface between PHD and 4HB; key residues mediating interactions through water molecules are shown in *stick* representation (*dotted lines*, polar interactions). *F*, interface between ZnF and PHD; key residues mediating interactions are shown in stick representation (*dotted lines*, polar interactions). 4HB, four-helix bundle.

We expressed a minimal fragment of *Arabidopsis thaliana* (*At*) VIN3 spanning its PHD signature (VIN3_150-208_) in *Escherichia coli* and purified this to homogeneity, but its crystallization was unsuccessful. We noticed that VEL PHD fingers are flanked by highly conserved sequences: their N-terminal flanking region contains four invariant cysteines, while their C-terminal flanks are predicted to be helical (Fig. 1*A*). We thus purified an extended fragment spanning the whole conserved region (VIN3_116-307_), but this proved to be toxic upon expression in *E. coli*. We next expressed the equivalent fragment from several other plants species and eventually succeeded in crystallizing the equivalent protein fragment (VIN3_123-326_) from the palm tree *Phoenix dactilyfera (Pd)*. VIN3_123-326_ produced diffracting crystals under multiple conditions, whereby the best crystals diffracted to 2.1 Å resolution in space group P3_1_21. We thus determined its structure by single wavelength anomalous dispersion using the intrinsic Zn^2+^ signals from ZnF and PHD (Table 1).

**Table 1:** Crystal data collection and refinement statistics

	*Pd*VIN3_123-326_
PDB accession code	7QCE
Crystal data	
Wavelength (Å)	0.97934
Resolution (Å)	50.78–2.1 (2.16–2.1)
Space group	P 3_1_ 2 1
Unit cell dimensions	
a, b, c (Å)	58.64, 58.64, 225.40
α, β, γ (°)	90.00, 90.00, 120.00
Total reflections	535,114 (45,246)
Unique reflections	27,349 (2183)
Multiplicity	19.6 (20.7)
Mean *I*/σ (*I*)	13.3 (2.7)
R merge (%)	13.5 (151.8)
CC _1/2_	0.997 (0.810)
Completeness (%)	100 (100)
Complexes in A.U	2
Refinement	
Resolution	50.78–2.1
Number of reflections	27,262 (2652)
Rwork/Rfree (%)	23.02–26.45
Nº of atoms	2701
Protein	2630
Ligand	19
Water	52
Average B Factors (Å^2^)	
Wilson/overall	43.73
Protein	36.99
Ligand	66.79
Water molecules	51.30
All atoms	37.37
RMSDs deviations	
Bond lengths (Å)	0.005
Bond angles (°)	1.28
Ramachandran plot Statistics (%)	
Favored regions	98.81
Allowed regions	1.19
Disallowed regions	0

Statistics for the highest-resolution shell are shown in parentheses.

VIN3_123-326_ forms a single compact domain, with an N-terminal ZnF and a C-terminal 4HB, packing tightly against the central PHD module (Fig. 1*B*). The topology of the α-helices in 4HB is up-down-up-down, with each helix packing in an antiparallel fashion against the preceding one, thereby forming two straight helical hairpins that associate with each other around a hydrophobic core (Fig. 1, *A*, *C*, and *D*). These cores are stabilized by Zn^2+^ ions (one in ZnF and two in PHD) that are coordinated by four conserved cysteines or by three cysteines and a histidine in the case of the first Zn^2+^ of the PHD finger (Fig. S1). This is the characteristic Cys4-His-Cys3 signature motif that defines classical PHD fingers (2, 3). The interface between ZnF and PHD finger is relatively small and exhibits only a small number of interactions mediated by water molecules (Fig. 1*E*), while that between PHD and 4HB is more extensive and formed by conserved residues engaging in multiple hydrophobic and polar interactions (Fig. 1*F*). Notably, one of the key interacting residues in the latter interface is Phe_216_ (Fig. 1*F*), the aromatic residue upstream of the final two zinc-coordinating cysteines, which constitutes part of the PHD signature (2, 3). Interestingly, its equivalents in classical PHD fingers, such as those from the Wnt signaling protein Pygo or from the lysine methyltransferase MLL1, are crucial in mediating interactions with nonhistone ligands (17, 26). This suggests a common role for this aromatic PHD signature residue in mediating *cis* or *trans* interactions with nonhistone protein modules.

The domain organization and sequence conservation of the tripartite superdomain structure of VIN3_123-326_ implies that the same superdomain would be present in the other VEL paralogs, a notion confirmed by structural predictions by AlphaFold (27). We note that there are precedents of PHD fingers forming superstructures with flanking domains, for example, with bromodomains in the KAP1 corepressor and in several TRIM proteins (28, 29, 30, 31) but also with a chromodomain in MLL1 (17) or with a ZnF module in the ubiquitin ligase UHRF1 (32). However, our searches of known protein structures confirmed that the tripartite architecture of the VIN3_123-326_ superdomain with a PHD finger at its core and flanking ZnF and 4HB domains is unique and unprecedented.

### A closely related PHD superdomain is found in OBERON proteins

**Figure 2:**
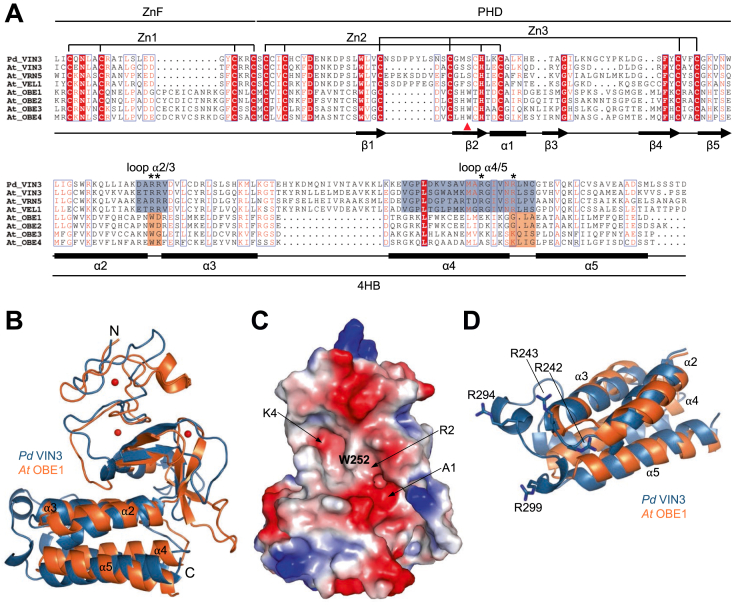
**The PHD superdomain fold is conserved in OBERON proteins.***A*, Sequence alignment between *P. dactylifera* (*Pd*) VIN3_134-326_, *A. thaliana (At)* VEL proteins (*At* VIN3_128-307_, *At* VRN5_47-239_, *At* VEL1_144-334_) and *At* OBE proteins (OBE1_195-368_, OBE2_196-373_, OBE3_406- 603_, OBE4_805-980_), with predicted OBE secondary structure indicated below; highlighted are the Zn^2+^-ligating residues of ZnF and PHD; *red triangle*, aromatic pocket-divider residue in OBE proteins; *blue and orange boxes*, loops connecting α2/3 and α4/5 at distal end of 4HB, with arginine quadruplet (R242, R243, R294, R299) indicated with an *asterisk*. *B*, superposition of ribbon representations of *Pd* VIN3_123-326_ crystal structure (*blue*) and corresponding structure of OBE1 predicted by AlphaFold (*orange*), with N and C termini indicated; *red balls*, zinc ions; RMSD 3.13 Å. *C*, molecular surface representation of *At* OBE1_195-368_ colored according to electrostatic potential (*red*, negative; *blue*, positive), same view as in (*B*), showing canonical PHD histone-binding surface (‘front’ face), with predicted A1, R2, and K4 pockets indicated by *black arrows*, and key aromatic residue between R2 and K4 pockets (W252, pocket-divider) labeled (see also Fig. S2). *D*, 4HB domain overlay of *Pd* VIN3_123-326_ and corresponding fragment of At OBE1, colored as in (*A*), with arginine quadruplet of VIN3 at the distal 4HB end in stick representation.

Given that the PHD superdomain fold of the VEL proteins is unique amongst known protein structures, we asked whether this fold might be present in proteins whose structure has not yet been determined. We therefore conducted a DALI search of protein structures predicted by AlphaFold (27), which identified a closely related PHD superdomain fold in a family of proteins called OBERON (OBE), comprising four paralogs in Arabidopsis. OBE proteins function in shoot and root meristems to control the growth of Arabidopsis plants, apparently by controlling the expression of a wide range of auxin-responsive genes (22, 23, 24, 25). Sequence comparison between VEL and OBE proteins confirms that the spacing of the three subdomains (ZnF, PHD, and 4HB) is highly conserved between the two families, as are the positions of the zinc-coordinating cysteines and histidines (Fig. 2*A*). Furthermore, the predicted fold of the OBE PHD superdomain is closely related to that of the VEL proteins (Fig. 2*B*), with low RMSD values for their backbones. For example, the RMSD values between the PHD superdomains of VIN3 and OBE1 is 3.13 Å and that between their PHD finger modules 1.46 Å, although we note an additional β-strand in the PHD fingers of all OBE family members, not present in VEL proteins (Fig. 2, *A* and *B*, compare to Fig 1*A*). Interestingly, while the PHD fingers of VEL proteins lack an aromatic pocket divider residue between the K4 and R2 pocket, which is critical for binding to methylated H3 tail in classical histone-binding PHD modules, OBE PHD fingers contain a tryptophan in this position (Figs. 2*A* and S2). The latter also exhibit a three-pronged pocket architecture typical of histone-binding PHD fingers (5, 6, 17) (Fig. 2, *A* and *C*), which is not apparent in the PHD module of VIN3_123-326_ (see also later). This suggests that the OBE but not the VEL PHD fingers contain functional histone H3-binding pockets that may underpin their function in gene control.

**Figure 3:**
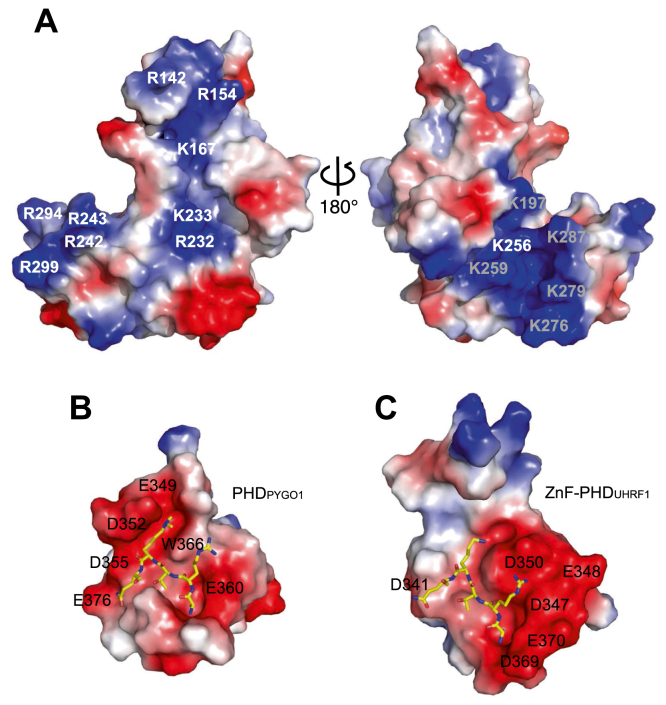
**The VIN3 PHD superdomain is positively charged.***A*, molecular surface representation of *Pd* VIN3_123-326_ showing electrostatic surface potential (*red*, negative; *blue*, positive), with highly conserved (*white*) and variable (*gray*) positively charged residues labeled. *B* and *C*, electrostatic surface potential as in (*A*), of canonical PHD fingers binding to H3K4me2 histone H3 peptides, with conserved negatively charged residues lining the histone H3-binding pockets labeled. *B*, PHD_PYGO1_ (PDB code 2VPE); (*C*) ZnF-PHD_UHRF1_ (PDB code 3SHB); *stick representations*, histone H3 peptides (*yellow*). PDB, Protein Data Bank.

Regarding the 4HB module, we note two main differences between VEL and OBE proteins. Firstly, the loops connecting individual α-helices are longer in VEL compared to OBE 4HB modules, whereby the loops between α2 and α3 are formed by five (VEL) instead of two residues (OBE), and those between α4 and α5 by 21 (VEL) instead of four residues (OBE), bulking out the distal end of the VEL 4HB modules, which thus tend to be larger than their OBE counterparts (Figs. 2 and 3*A*). The lengths of these loops at the distal 4HB ends are highly conserved within each family (Fig. 2*A*), pointing to functional significance. Notably, the long loops in the VEL proteins contain four highly conserved arginine residues (R242, R243, R294, and R299 in *Pd* VIN3) that are not present in their OBE counterparts (Fig. 2*A*). This arginine quadruplet forms a pronounced positively charged surface patch on the distal 4HB ends of VEL proteins (Fig. 3*A*).

### Conserved positive surface potential in VEL PHD superdomains

A striking feature of the VIN3 PHD superdomain is its extensive positive electrostatic surface potential. Indeed, positively charged surface patches are seen on both faces of the PHD superdomain, with one of these being located at the distal end of the 4HB module, as mentioned previously, and another extending across the PHD finger surface (Fig. 3*A*). The latter contrasts with the negative electrostatic surface potential typically found in canonical histone-binding PHD fingers (5, 6, 17) including that of human PYGO1 and the ZnF-PHD module of UHRF1 (Fig. 3, *B* and *C*). It is also distinct from the PHD finger surface of the OBE proteins whose overall surface potential is negative (Figs. 2*C* and S2), compatible with binding to the positively charged histone H3 tail.

**Figure 4:**
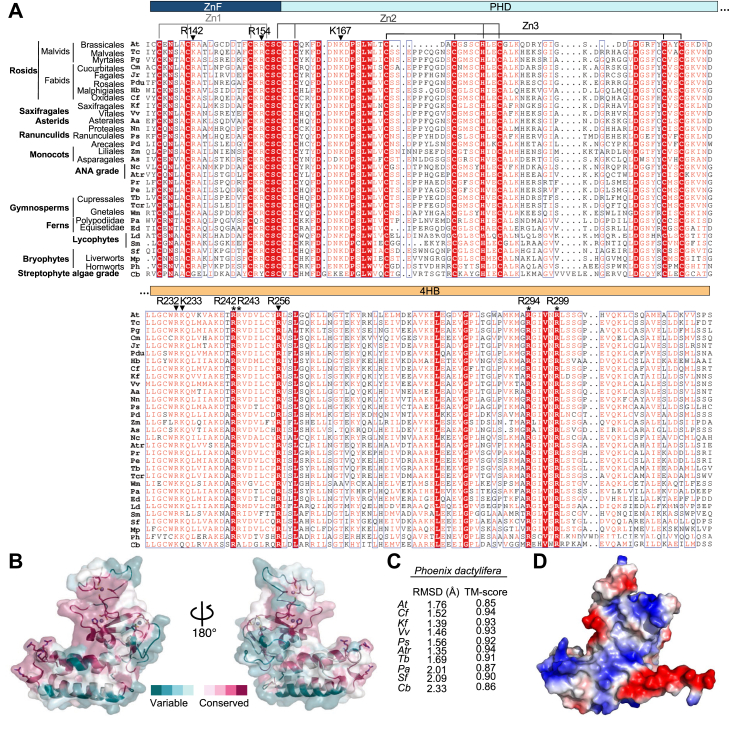
**Deep conservation of PHD superdomains in plants.***A*, sequence alignments of VEL PHD superdomains from diverse plant species as indicated, belonging to angiosperms (At, *Arabidopsis thaliana*; Tc, *Theobroma cacao*; Pg, *Punica granatum*; Cm, *Cucumis melo*; Jr, *Juglans regia*; Pdu, *Prunus dulcis*; Hb, *Hevea brasiliensis*; Cf, *Cephalotus folicularis*; Eg, *Elaeis guineensis*; Kf, *Kalanchoe fedtschenkoi*; Vv, *Vitis vinifera*; Aa, *Artemisia annua*; Nn, *Nelumbo nucifera*; Ps, *Papaver somniferum*; Pd, *Phoenix dactylifera*; Zm, *Zostera marina*; As, *Apostasia shenzhenica*; Nc, *Nymphaea colorata*; Atr, *Amborella trichopoda*; Pr, *Pinus radiata*; Pe, *Picea engelmanii*), gymnosperms (Tb, *Taxus baccata*; Tcr, *Taiwania cryptomerioides*; Wm, *Welwitschia mirabilis*), ferns and lycophytes (Pa, *Polystichum acrostichoides*; Ed, *Equisetum diffusum*; Ld, *Lycopodium deuterodensum*; Sm, *Selaginella moellendorffii*), bryophytes (Sf, *Sphagnum fallax*; Mp*, Marchantia polymorpha*; Ph, *Paraphymatoceros hallii*) or *algae* (Cb, *Chara braunii*); *white in red boxes*, invariant residues; *red in blue frames*, similar residues across clades. Indicated above sequences are Zn^2+^-ligating residues in ZnF and PHD; *arrowheads*, conserved residues that form the positively charged surface patches of the PHD superdomain; *asterisks*, the near-invariant arginine quadruplet within 4HB. *B*, conservation of surface-exposed residues as calculated by ConSurf (44), displayed on the semitransparent surface of *Pd* VIN3_123-326_, with degree of sequence conservation color coded as indicated in key. *C*, RMSD and TM-score values for VEL PHD superdomain structures from various species, abbreviated as in (*A*), relative to the *Pd* VIN3_123-326_ crystal structure. *D*, structure of VIN3 PHD superdomain from the alga *Chara braunii* predicted by AlphaFold, same view as in (*B*) left, showing conservation of distal arginine quadruplet in 4HB and of positively charged front surface equivalent to the histone binding-pocket of canonical PHD fingers. 4HB, four-helix bundle.

The positively charged surface patch extending across the *Pd* VIN3 PHD finger is formed by basic amino acids from each of the three subdomains (R142 and R154 of ZnF; K167 of PHD; R232 and K233 of 4HB; Fig. 1*A*). Notably, basic residues in these locations of the three subdomains are near-invariant across different species (Fig. 4*A*). The same is true for the positively charged distal patch in the 4HB module (Fig. 3*A*) whose contributing four basic residues (R242, R243, R294, and R299 in *Pd* VIN3) are found throughout the plant kingdom, from flowering plants all the way to bryophytes (Fig. 4*A*). ConSurf analysis confirmed that the positive surface patches on the front face of the PHD superdomain are deeply conserved across species, coinciding with areas of highest conservation (Fig. 4*B*).

To determine whether these deeply conserved positively charged residues of VEL PHD superdomains also form contiguous surface patches in PHD superdomains of distant species, we conducted AlphaFold structure predictions of these domains from angiosperms to algal species. Indeed, this confirmed that the backbones of the predicted structures are highly similar to our VIN3_123-326_ structure determined for the Phoenix protein, exhibiting low RMSD and high template-modeling (TM) score values between the experimentally determined structure and the AlphaFold predictions for other VEL PHD superdomains. In particular, the TM scores resulting from comparisons between different species range from 0.85 to 0.94 (Fig. 4*C*), whereby 1 indicates a perfect match between two structures. For example, the predicted fold of the PHD superdomain of the alga *Chara braunii* (Fig. 4*D*) closely resembles that of our crystal structure for the palm tree domain (Fig. 3*A*). This close resemblance of the folds amongst the different species allowed us to confirm that the deep conservation extends to the positively charged surface patch across the PHD finger and that located at the distal end of the 4HB subdomain, even though one of the arginines in the arginine quadruplet that constitutes this patch is missing in this algal species (Fig. 4*A*). We conclude that the front face of the VEL PHD superdomain is highly likely to exhibit positive surface potential even in evolutionary distant species, which suggests functional relevance of this striking feature, for example, in ligand binding.

### The VEL PHD superdomain is not a histone H3 reader

**Figure 5:**
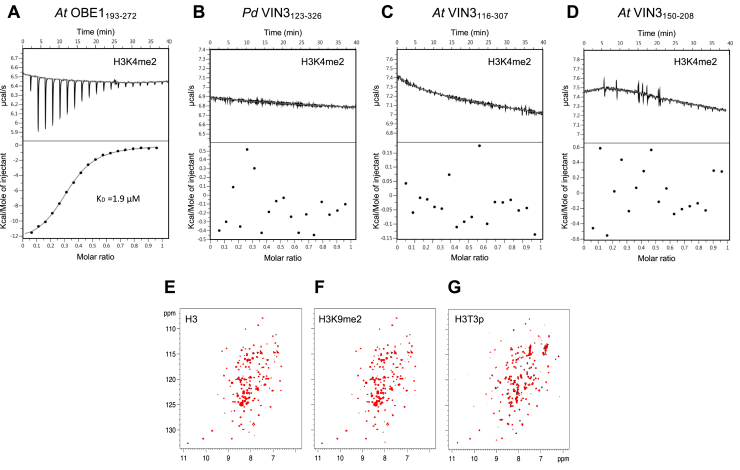
**The PHD superdomain of OBE1 but not of VIN3 binds to histone H3 tail peptides.***A*–*D*, representative ITC profiles of (*A*) *At* OBE1_193-372_, (*B*) *Pd* VIN3_123-326_, (*C*) *At* VIN3_116-307_, or (*D*) *At* VIN3_150-208_ incubated with H3K4me2, revealing binding of methylated histone H3 tail in the low micromolar range (*K*_*D*_ 1.9 μM) in case of the OBE PHD superdomain, whereas binding cannot be detected for the VIN3 PHD superdomain nor for its minimal PHD finger module. *E*–*G*, overlays of BEST-TROSY spectra of 50 μM ^15^N-labeled *Pd* VIN3_123-326_, alone (*black*) or incubated with 0.5 mM of unmodified H3 21-mer (*red*), H3K9me2 15-mer (*red*), or H3T3p 15-mer (*red*) as indicated in panels.

ITC is commonly used to monitor binding of histone H3 tail peptides to PHD fingers, which typically exhibit affinities in the low micromolar range (6, 17). Since we expected the OBE PHD superdomain to bind to H3K4me peptides, based on its architecture and negative surface charge, we used ITC to assay purified PHD superdomain from Arabidopsis OBE1 (OBE1_193-272_) for its binding to H3K4me2. This revealed a low micromolar affinity (*K*_*D*_ 1.9 μM; Fig. 5*A*), similar to that determined for classical histone H3 tail-binding PHD fingers (6, 17). By contrast, we were unable to detect binding of purified PHD superdomain from Phoenix (*Pd* VIN3_123-326_) to H3K4me2 (Fig. 5*B*) nor to other histone H3 tail peptides (methylated or unmodified; Table 2), including H3K9me2, which was previously reported to be strongly positive for the Arabidopsis PHD domain (20, 21) (see also later). Indeed, probing a modified histone peptide array (MODified Histone Peptide Array, Active Motif) with this PHD superdomain yielded entirely negative results (see Experimental Procedures). We also tested the purified orthologous PHD superdomain from Arabidopsis (VIN3_116-307_) after expression in insect cells and a minimal protein fragment spanning its PHD finger alone (VIN3_150-208_) whose expression in *E. coli* yielded soluble protein, but neither of these Arabidopsis proteins exhibited binding to H3K4me2 (Fig. 5, *C* and *D*).

**Table 2:** Histone H3 peptides tested for binding to various PHD-containing modules

Construct Peptide	*Pd* VIN3_123-326_	*At* VIN3_116-307_	*At* VIN3_150-208_	*At* VIN3_130-299_	*At* OBE1_193-272_
Unmodified H3 (1–21)	ITC & NMR	ITC	ITC & NMR	−	−
H3K4me2 (1–15)	ITC & NMR	ITC	ITC & NMR	ITC	ITC
H3K9me2 (1–15)	ITC & NMR	ITC	ITC & NMR	−	−
H3T3p (1–15)	ITC & NMR	ITC	ITC & NMR	−	−
H3S10p (1–15)	ITC & NMR	ITC	ITC & NMR	−	−

Summary of histone H3 peptides tested with ITC or NMR in this study for binding to *Pd* or *At* PHD superdomains or to the minimal *At* VIN3 PHD finger, each with negative outcome.

Noting that some PHD fingers have low affinities for histone H3 tails (12), we turned to NMR spectroscopy, a highly sensitive technique capable of monitoring weak interactions in the millimolar *K*_*D*_ range (33) to test the binding of the same peptides to ^15^N-labeled VIN3_123-326_. However, there was no detectable perturbation of its BEST-TROSY spectra for any of three peptides (Figs. 5, *E* and *F* and S3). Given the positive surface potential of VIN3_123-326_, we also tested histone H3 peptides phosphorylated at threonine 3 or serine 10 (H3T3p, H3S10p) since phosphorylation of these histone H3 tail residues has been detected in animals and plants (34, 35). However, binding of VIN3_123-326_ to these peptides proved to be equally negative in both NMR (Figs. 5*G* and S3) and ITC (E. F.-E., unpublished). We regard this as conclusive evidence that the PHD superdomain of VIN3 does not bind to histone H3 tails.

Our results are in contrast with the previous evidence for the binding of the same histone tail peptides to the minimal PHD modules of several VEL paralogs (20, 21). In an attempt to reconcile our data with the previous reports, we purified the VIN3 PHD superdomain from the plasmid provided by Sung and colleagues (encoding amino acids 130–299 from *A. thaliana* VIN3), following their protocol that involved preparing total unfractionated His-tagged protein by Ni-NTA affinity chromatography after expression in *E. coli*. However, we could not detect any binding of this protein to H3K4me2 by ITC (Fig. S4). We noted that the protein encoded by this plasmid includes a C-terminal extension of 18 aa of unknown origin. Furthermore, when examining the Ni-NTA–eluted protein by polyacrylamide electrophoresis, we found that only a minor fraction of this eluate corresponded to full-length domain, and mass spectrometry indicated that the two main breakdown products corresponded to the maltose-binding protein (MBP) tag alone or to a C-terminally truncated PHD superdomain domain without the last two alpha-helices of the 4HB domain (Fig. S4). This C-terminally truncated protein fragment may be partially unfolded and might therefore have trapped histone H3 peptides in pull-down assays, which could explain the apparently positive results reported previously (20, 21).

### The PHD superdomain of VIN3 has a low affinity for negatively charged polymers

**Figure 6:**
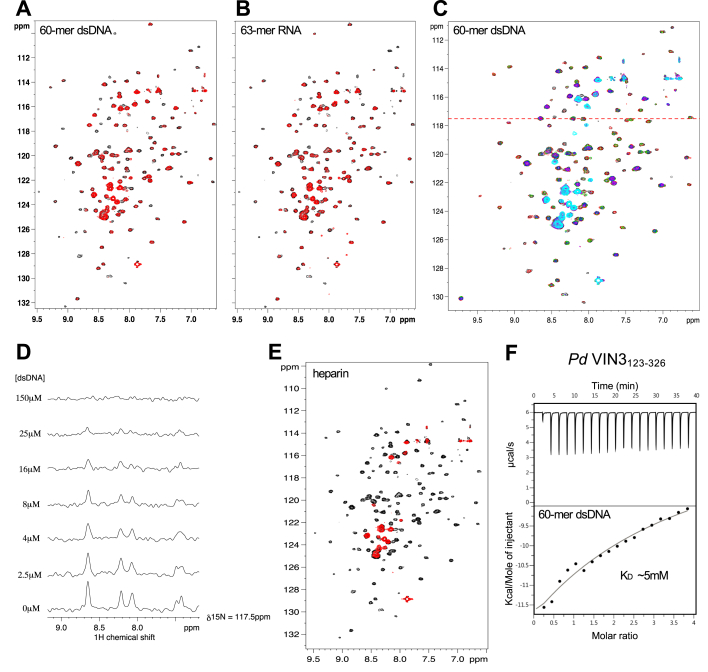
**Interaction of PHD superdomain with negatively charged polymers.***A* and *B*, overlays of BEST-TROSY spectra of 50 μM ^15^N-labeled *Pd* VIN3_123-326_, alone (*black*) or incubated with (*A*) 4 μM 60-mer dsDNA (*red*) or (*B*) 4 μM 63-mer RNA (*red*). *C*, overlays of BEST-TROSY spectra of 50 μM ^15^N-labeled VIN3_123-326_, alone (*black*) or titrated with 60-mer dsDNA (*red*, 2.5 μM; *green*, 4 μM; *blue*, 8 μM; *pink*, 16 μM; *purple*, 25 μM; *cyan*, 150 μM). *D*, slices corresponding to titrations in (*C*), depicting height changes of selected crosspeaks, taken from the BEST-TROSY spectra of *Pd* VIN3_123-326_ at the position indicated by the *red dashed line* in (*C*). *E*, overlays of BEST-TROSY spectra of 50 μM ^15^N-labeled *Pd* VIN3_123-326_, alone (*black*) or incubated with 4 μM 60-mer heparin (*red*). *F*, representative ITC profile of *Pd* VIN3_123-326_ incubated with 60-mer ssDNA, revealing weak binding in the low millimolar range (*K*_*D*_ ∼ 5 mM).

Given the conserved positive surface potential of the VEL PHD superdomains, we wondered whether these domains might be able to bind to nucleic acids. We therefore used NMR to test whether ^15^N-labeled VIN3_123-326_ would interact with DNA or RNA oligomers. Given the observed association of VEL proteins with the nucleation region of *FLC* (18, 19), we chose the core sequence from this region as a template to generate single- or double-stranded 60-mer DNA or a 63-mer RNA transcript. Incubating these probes with purified ^15^N-labeled VIN3_123-326_ revealed that both RNA and dsDNA samples produced perturbations of the same individual crosspeaks of its BEST-TROSY spectrum (Fig. 6, *A* and *B*), with ssDNA less effective in eliciting spectral changes than dsDNA (E. F.-E., unpublished). The minimal concentration of dsDNA (or RNA) required to produce spectral perturbations was 2 μM, but titrations with gradually increasing concentrations of dsDNA (or RNA) produced more pronounced spectral changes, culminating in a widespread broadening of crosspeaks upon incubation with 150 μM dsDNA (Fig. 6, *C* and *D*). These observations suggest that the VIN3 PHD superdomain has a moderate propensity for interacting with dsDNA and RNA. We also found the same set of perturbations of the BEST-TROSY spectrum of ^15^N-labeled VIN3_123-326_ upon incubation with 4 μM heparin (Fig. 6*E*), a negatively charged helical polymer consisting of sulfated disaccharide subunits. This strongly suggests that the observed interactions with DNA and RNA are nonsequence specific. Next, we used ITC titrations to confirm a weak affinity in the low millimolar range (*K*_*D*_ ∼ 5 mM) between purified *Pd* VIN3_123-326_ and dsDNA 60-mer (the only nucleic acid probe we were able to generate at sufficiently high concentration). Taken together, our experiments provide evidence for an inherent propensity of VIN3_123-326_ to interact weakly with negatively charged polymers, presumably owing to its positive surface potential. It reinforces the notion that the physiological ligands for the PHD superdomain of VEL proteins are likely to be characterized by a negative surface potential.

## Discussion

Our crystal structure of a VIN3 protein fragment encompassing its PHD finger revealed that this finger is the central module of a larger tripartite PHD superdomain conserved in all VEL proteins. This PHD superdomain fold is found in only one other protein family, the OBE proteins that control plant growth through regulation of gene expression in root and shoot meristems (22, 23, 24, 25). However, unlike the OBE PHD subdomain whose structure is predicted to exhibit the typical three-pronged pocket architecture of histone-binding PHD fingers with overall negative surface potential (4, 16, 36) (Fig. 2*C*), the PHD fingers of the VEL proteins lack this pocket architecture and show positive surface potential (Figs. 3*A*, 4*D*, and S2). It was therefore unsurprising that we were unable to detect an interaction between the VIN3 PHD superdomain and variously modified or unmodified histone H3 peptides (Figs. 5, S3 and Table 2). As far as we are aware, this represents the first example of a PHD finger for which there is conclusive evidence that it does not bind to histone H3 tails. We therefore regard it as highly unlikely that the VEL proteins function as histone code readers (20, 21).

However, the positively charged surface patches of the VIN3 PHD superdomain are highly conserved amongst VEL orthologs throughout the plant kingdom (Figs. 3*A*, 4, *A*,and *B*), which indicates their functional relevance for ligand binding. In support of this, we obtained evidence by NMR and ITC that the VIN3 PHD superdomain has an intrinsic propensity to interact weakly with negatively charged polymers such as RNA and DNA (Fig. 5). This raises the possibility that the physiological ligand of the VEL PHD superdomain involves DNA at the *FLC* Polycomb Response Element or regulatory noncoding RNAs COOLAIR or COLDWRAP (37, 38). It is also conceivable that this domain uses its arginine quadruplet as an anchor to engage the acidic patch of the H2A-H2B dimer on the nucleosome surface, as described for other chromatin complexes (39). Regardless of whether any of these apply, our work has highlighted that the VEL PHD superdomains have evolved as positively charged structural platforms that serve as an interface for binding to negatively charged nonhistone tail ligands.

## Experimental procedures

### Protein expression and purification

VIN3_PHD_ (residues 150–208) complementary DNA from *A. thaliana* was inserted into pETM-41 containing a hexahistidine tag followed by an MBP tag and a 3C protease cleavage site at the N terminus. VIN3_150-208_ was expressed in BL21 CodonPlus(DE3)-RIL cells (Agilent) in LB medium. Cells were grown at 37 °C to *A*_600_ 0.6, then moved to 18 °C, followed by induction with 0.4 mM IPTG and 100 μM ZnCl_2_ at *A*_600_ 0.8. Harvested cells were resuspended in lysis buffer (25 mM Tris pH 8.0, 200 mM NaCl, 20 mM imidazole pH 8, and EDTA-free protease inhibitor cocktail (Roche)) and lysed by sonication (Branson). Cleared lysates were loaded onto Ni-NTA resin (Qiagen) by gravity flow and washed with lysis buffer. After extensive washing, samples were eluted with lysis buffer supplemented with 300 mM imidazole. Eluted samples were incubated with 2 mM DTT and cleaved by 3C protease (made in house; protease:protein ratio 1:80) overnight at 4 °C. Eluted proteins were further purified by anion-exchange chromatography (monoQ, GE Healthcare) and finally loaded onto a HiLoad 26/600 Superdex 75 pg column (GE Healthcare) equilibrated in 25 mM Tris pH 8, 150 mM NaCl, and 1 mM DTT. Peak fractions were pooled and concentrated for crystallization assays.

VIN3_116-307_ complementary DNA from *A. thaliana* was inserted into a pFastBac vector (Invitrogen) containing a hexahistidine tag followed by an MBP tag and a 3C protease cleavage site at the N terminus. Bacmids of this construct were obtained using the DH10EMBacY kit (Geneva Biotech). Baculovirus was generated using FuGENE HD Transfection reagent (Promega) in Sf9 cells using Insect X-press (Lonza) medium and infected cells were grown for 60 to 72 h. Harvested cells were lysed and purified as described previously using Ni-NTA resin (Qiagen). Tags were cleaved with 3C protease and incubated overnight by gentle rocking at 4 °C, followed by purification on a HiLoad 26/600 Superdex 200 pg column (GE Healthcare) in 25 mM Mes pH 6, 200 mM NaCl, and 1 mM DTT.

VIN3_123-326_ from *P. dactilyfera* was amplified by PCR from synthetic codon optimized genes (gBlocks, Integrated DNA Technologies) and subcloned into pEC-LIC-His-3C (kindly provided by Jurg Muller) containing a hexahistidine tag and a 3C protease cleavage site at the N terminus. VIN3_123-326_ was expressed and purified as described previously for VIN3_150-208_. In this case, after 3C cleavage overnight, the sample was loaded on a HiLoad 26/600 Superdex 200 pg column (GE Healthcare) in 25 mM Tris pH 8, 150 mM NaCl, and 1 mM DTT.

OBE1_193-372_ from *A. thaliana* was amplified by PCR from synthetic codon-optimized genes (gBlocks, Integrated DNA Technologies) and inserted into pEC-LIC-His-3C. OBE1_193-372_ expression was done as described previously for VIN3_150-208_. Harvested cells were lysed and purified, as described, after Ni-NTA chromatography and 3C protease cleavage overnight, and OBE1_193-372_ was purified after fractionation with a HiLoad 26/600 Superdex 200 pg column (GE Healthcare) in 25 mM Tris pH 8, 150 mM NaCl, and 1 mM DTT.

For the purification of S-Tag-6×His-MBP-VIN3_130-299_ used in Fig. S3, pVP13 (20, 21) kindly provided by Sibum Sung was used for protein expression at 18 °C overnight, after induction with 0.4 mM IPTG and supplementation with 100 μM ZnCl_2_. Harvested cells were lysed and purified by Ni-NTA affinity chromatography as described previously for VIN3_150-208_. Ni-NTA–eluted material was dialyzed into 25 mM Tris pH 8, 150 mM NaCl, and 1 mM DTT, and ITC titrations were performed as described.

### Protein crystallization, data collection, and structure determination

Pure fractions of VIN3_PHD_, VIN3_123-326_, and VIN3_116-307_ were concentrated with a 3 or 10 kD MWCO Vivaspin 20 concentrator (Sartorius) to 10 to 35 mg ml^−1^. Crystallization trials with multiple commercial crystallization kits were performed in 96-well sitting-drop vapor diffusion plates (Molecular Dimensions) at 18 °C and set up with a mosquito HTS robot (TTP Labtech). VIN3_123-326_ crystals emerged under multiple conditions after growing for several days by the vapor diffusion method. Prior to data collection, all crystals were transferred for a few seconds to the crystallization solution plus 25% (v/v) glycerol and then flash frozen in liquid nitrogen. Diffraction data for VIN3_123-326_ were collected at the Diamond Light Source synchrotron (beamline I03) from crystals grown in 0.06 M Morpheus Divalents, 1.1 M Morpheus Buffer System pH 6.5, 10% PEG 20K, and 20% PEG 500 MME.

Data processing was performed with XIA2 DIALS and scaled using Aimless from CCP4 (Collaborative Computational Project, Number 4, 1994). VIN3_123-326_ was solved by single-wavelength anomalous dispersion technique using SHELX (40). The structure was built with ArpWarp (41) and model refinement was performed by alternating cycles of automatic refinement with REFMAC (42) and manual building with COOT (43). Structural images were prepared with PyMol (version 1.8 Schrödinger, http://www.pymol.org/). The degree of evolutionary conservation of positive and negative surface charges was calculated by ConSurf (44).

### ITC measurements

ITC was carried out at 25 °C with an iTC 200 Microcalorimeter (GE Healthcare) in 25 mM Tris pH 8, 150 mM NaCl, and 1 mM DTT. Titrations consisted of 19 consecutive 2 μl injections of 0.25 to 1 mM peptide solution (following a preinjection of 0.5 μl) into 50 μM protein (VIN3_PHD_, VIN3_123-326_, VIN3_116-307,_ OBE1_193-372_, or pVP13 VIN3_130-299_) at time intervals of 120 s. ITC data were analyzed using MicroCal PEAQ-IT Analysis Software (Malvern Sciences).

### NMR spectroscopy

For NMR spectroscopy, *A. thaliana* VIN3_150-208_ and *P. dactilyfera* VIN3_123-326_ were expressed in M9 minimal medium supplemented with 0.4% glucose, antibiotics, trace elements, 25 ml overnight culture, and 2 g of ^15^N-H_4_Cl per liter of expression culture. Cultures were grown and processed essentially as described before. NMR samples containing 50 μM ^15^N-labeled VIN3_150-208_ or VIN3_123-326_ were prepared in aqueous buffer (25 mM Tris pH 8, 150 mM NaCl, and 1 mM DTT). The 50 μM ^15^N-labeled VIN3_150-208_ or VIN3_123-326_ was incubated with 0.5 mM histone H3 peptides resuspended in 25 mM Tris pH 8, 150 mM NaCl, and 1 mM DTT for >30 min before measurement. Spectra were recorded on a Bruker Avance-III spectrometer operating at 600 MHz ^1^H frequency and equipped with a 5 mm inverse detection cryogenic probe. BEST-TROSY shift correlation experiments (45) were acquired with 256 × 1024 points in *t*_1_ and *t*_2_, respectively. Spectra were processed in TopSpin version 3.2 (Bruker), with linear prediction in *t*_1_, giving a digital resolution of 2.2 and 8.2 Hz/point in the processed data.

### Probing of histone H3 peptide array

A histone H3 peptide array (MODified Histone Peptide Array, Active Motif) was carried out with purified *Pd* VIN3_116-307_ containing a hemagglutinin (HA)-tag at its C terminus. Bacmids, baculovirus, and expression and purification of VIN3_116-307_-HA were produced as described previously. After blocking and incubation of the array with VIN3_116-307_-HA, detection was performed with an anti-HA tag antibody (Abcam, catalog no.: #ab9110) and horseradish peroxidase–conjugated secondary antibody (Invitrogen, Cat#A16124) on film.

### Generation of nucleic acid probes for NMR

*FLC* DNA was used as a template for the design of a dsDNA 60-mer probe, generated by annealing the sequence (5′ GCGGTACACGTGGCAATCTTGTCTTCAAAACACAACGTTTTTATTCACATATTTGGTTTT 3′) and its complementary oligonucleotide for 2 min at 95 °C. For the ssDNA probe, an alternative 60-mer sequence of *FLC* predicted to lack secondary structure (5′ GAAGAAAAAGTAGATAGGCACAAAAAATAGAAAGAAATAAAGCGAGAAAAGGAAAAAAAA 3′) was selected as template. For the generation of RNA probes, a 60-mer fragment from *FLC* predicted to form R-loops (5′ *GGG*AAAGAGAAAACGCUUAGUAUCUCCGGCGACUUGAACCCAAACCUGAGGAUCAAAUUAGGG 3′) was selected for amplification by PCR. A T7 promoter region was inserted upstream of this sequence, and a dsDNA PCR product was used as template for *in vitro* transcription with the HiScribe T7 High Yield RNA Synthesis Kit.

### Phylogenetic analysis and protein sequence alignment

Protein sequences of VEL orthologs were obtained from the OneKP consortium (www.onekp.com) or retrieved from JACKHMMER (https://www.ebi.ac.uk/Tools/hmmer/search/jackhmmer). Alignments of sequences were done with MacVector (MacVector Inc) and ESPRIPT using the ClustalW algorithm and Clustal Omega, respectively.

## Data availability

The accession number for the coordinates and structure factors reported in this study is PDB 7QCE.

## Supporting information

This article contains supporting information.

## Conflict of interest

The authors declare that they have no conflicts of interest with the contents of this article.
