# Supplementary material for: Plant vernalization proteins contain unusual PHD superdomains without histone H3 binding activity
Source: J Biol Chem. 2022 Sep 27;298(11):102540. doi: 10.1016/j.jbc.2022.102540 (PMC9640981; doi:10.1016/j.jbc.2022.102540)
Supplement: Supplemental information [file mmc1.pdf]

Supporting information for

Plant VEL proteins contain unusual PHD superdomains without histone H3 binding activity

Elsa Franco-Echevarría, Trevor J. Rutherford, Marc Fiedler, Caroline Dean & Mariann Bienz

This document includes:

Fig S1. Zinc-binding sites of the VIN3 PHD superdomain
Fig S2. Sequence alignment of various PHD fingers
Fig S3. The VIN3 PHD finger does not bind to histone H3 tail
Fig S4. Purification of S-Tag-6xHis-MBP-$VIN3_{130-299}$

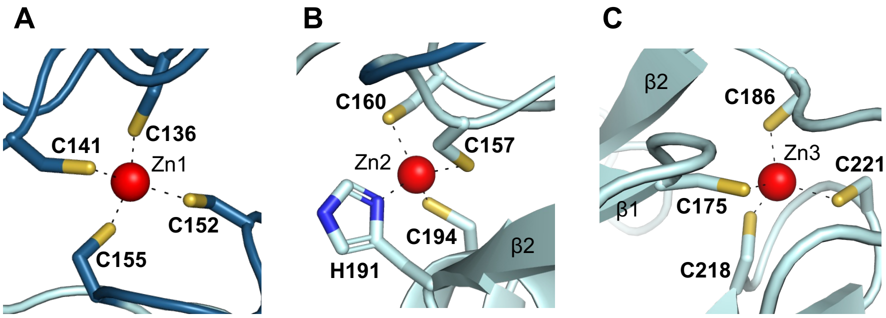

Figure S1. Zinc-binding sites of the VIN3 PHD superdomain. (**A-C**) Schematic representations of three Zn^{2+}-coordinating sites in *Pd* VIN3 superdomain in ZnF (Zn1) and PHD finger (Zn2 and Zn3); coordinating residues are shown in stick; *red balls*, Zn^{2+} ions.

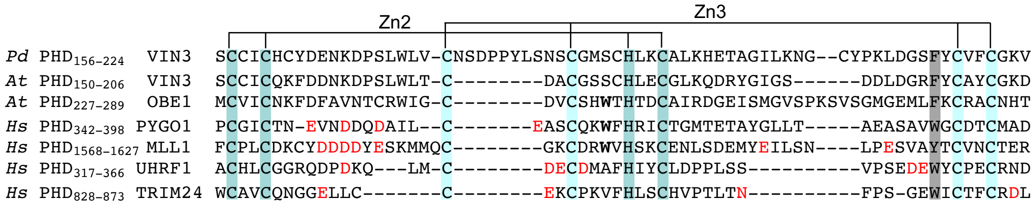

Figure S2. Sequence alignment of various PHD fingers. Sequence alignment of *Pd* and *At* VIN3 PHD fingers with H3K4me-binding PHD fingers, as indicated on the left (*Hs, homo sapiens*), with negatively-charged residues lining the histone-binding pockets in red; *bold*, tryptophane pocket divider residue (between K4 and A2 pockets) in H3K4me-binding PHD fingers; highlighted are Zn^{2+}-ligating residues (Zn2, *cyan*; Zn3, *light blue*) and aromatic PHD signature residue (*gray*).

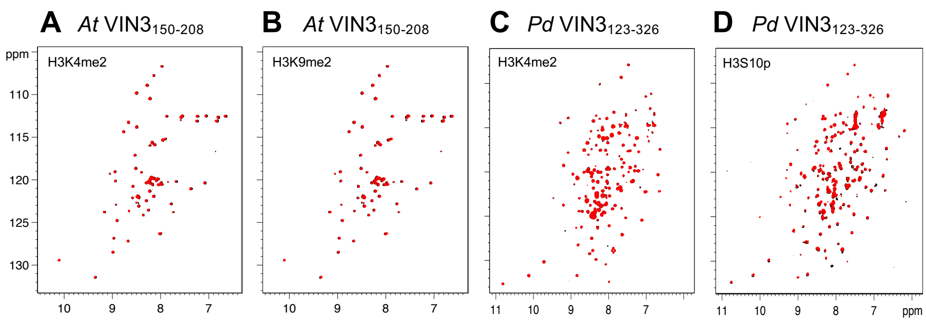

Figure S3. The VIN3 PHD finger does not bind to histone H3 tail. (**A, B**) Overlays of BEST-TROSY spectra of 50 mM ^{15}N-labeled *At* VIN3$_{150\text{-}208}$, alone (*black*) or incubated with 0.5 mM of H3K4me2 15-mer (*red*) or H3K9me2 15-mer (*red*) as indicated in panels. (**C, D**) Overlays of BEST-TROSY spectra of 50 mM ^{15}N-labeled *At* VIN3$_{123\text{-}326}$, alone (*black*) or incubated with 0.5 mM of H3K4me2 15-mer (*red*) or H3S10p 15-mer (*red*) as indicated in panels.

A MGKETAAAKFERQHMDSPPHHHHHHSKIEEGKLVIWINGDKGYNGLAEVGKKFEKDTGIKVTVEHPDKLEEKFPQVAATGDGPDIIFWAHDRFGGYAQSGLLAEITPDKAFQDKLYPFTWDAVRYNGKLIAYPIAVEALSLIYNKDLLPNPPKTWEEIPALDKELKAKGKS
ALMFNLQEPYFTWPLIAADGGYAFKYENGKYDIKDVGVDNAGAKAGLTFLVDLIKNKHMNADTDYSIAEAAFNKGETAMTINGPWAWSNIDTSKVNYGVTVLPTFKGQPSKPFVGVLSAGINAASPNKELAKEFLENYLLTDEGLEAVNKDKPLGAVALKSYEEELAKDP
RIAATMENAQKGEIMPNIPQMSAFWYAVRTAVINAASGRQTVDEALKDAQTNSSSNNNNNNNNNNLGIDENLYLTSLYKKAGSAAAPFTCENLACRAALGCDDTFCRRCSCCICQKFDDNKDPSLWLTCDACGSSCHLECGLKQDRYGIGSDDLDGRFYCAYCGKDND
LLGCWRKQVKVAKETRRVDVLCYRLSLGQKLLRGTTKYRNLLELMDEAVKKLEGDVGPLSGWAMKMARGIVNRLSSGVHVQKLCSQAMEALKGGRADPAFLYKVVMARL

B

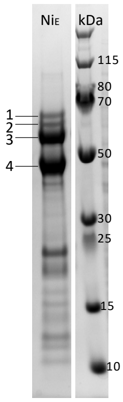

C

	SAMPLE 1	SAMPLE 2	SAMPLE 3	SAMPLE 4
ZnF	AALGCDDTFCR^{3}<br>AALGCDDTFCRR^{2}	AALGCDDTFCR^{5}<br>AALGCDDTFCRR^{2}	AALGCDDTFCR^{33}<br>AALGCDDTFCRR^{4}	AALGCDDTFCR
	CSCCICQK^{1}	CSCCICQK^{3}<br>RCSCCICQK^{1}	CSCCICQK^{5}<br>RCSCCICQK^{2}	
PHD	FDDNKDPSLWLTCDACGSSCHLECGLK^{1}	DPSLWLTCDACGSSCHLECGLK^{2}<br>FDDNKDPSLWLTCDACGSSCHLECGLK^{6}	DPSLWLTCDACGSSCHLECGLK^{4}<br>FDDNKDPSLWLTCDACGSSCHLECGLK^{57}	FDDNKDPSLWLTCDACGSSCHLECGLK^{1}
	YGIGSDDLDGR^{10}	YGIGSDDLDGR^{9}	YGIGSDDLDGR^{31}<br>YGIGSDDLDGRFYCAYCGK^{1}<br>QDRYGIGSDDLDGR^{1}	YGIGSDDLDGR^{2}
	FYCAYCGK^{5}	FYCAYCGK^{10}<br>FYCAYCGKDNDLLGCWR^{1}	FYCAYCGK^{35}<br>FYCAYCGKDNDLLGCWR^{2}	FYCAYCGK^{3}
	DNDLLGCWR^{6}		DNDLLGCWR^{32}<br>DNDLLGCWRK^{1}	DNDLLGCWR^{3}
4HB	VDVLCYR<br>RVDVLCYR^{5}	VDVLCYR^{2}<br>RVDVLCYR^{13}	VDVLCYR^{4}<br>RVDVLCYR^{9}	RVDVLCYR^{2}
	NLLELMDEAVK^{10}<br>NLLELMDEAVKK^{4}	NLLELMDEAVK^{31}<br>NLLELMDEAVKK^{6}	NLLELMDEAVK^{1}	NLLELMDEAVK^{1}
	KLEGDVGPLSGWAMK^{9}<br>LEGDVGPLSGWAMK^{1}	KLEGDVGPLSGWAMK^{7}<br>LEGDVGPLSGWAMK^{3}		
	LSSGVHVQK^{2}	LSSGVHVQK^{1}		

D

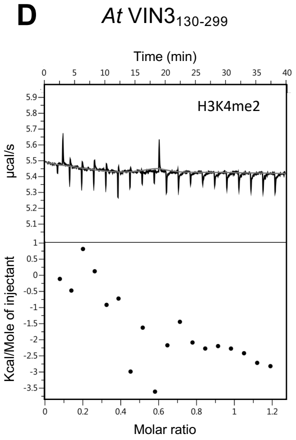

Figure S4. Purification of S-Tag-6xHis-MBP-VIN3$_{130\text{-}299}$. (**A**) Sequence of pVP13 (20,21) encoding *At* VIN3 PHD superdomain (S-tag-6xHis-MBP-VIN3$_{130\text{-}299}$), with sequences of ZnF, PHD and 4HB modules colored as in **Fig. 1**; *red*, S- and 6xHis-tags; *gray*, MBP tag plus C-terminal linker; *black*, additional 18 residues of unknown origin at the C-terminus of 4HB module. (**B**) Analysis of Ni-NTA-eluted material (Ni_E) by polyacrylamide gel electrophoresis, alongside molecular weight (MW) markers run on the same gel, revealing a minor fraction of full-length PHD superdomain (predicted MW 68.2 kDa) in addition to two major breakdown products. (**C**) Mass spectrometry results obtained from four products (1-4), as labeled in (**B**); peptide counts and origins of corresponding domains are indicated in table. (**D**) ITC profile of *At* VIN3$_{130\text{-}299}$ incubated with H3K4me2 15-mer, revealing lack of binding.
